# Electroretinographical analysis of the effect of cannabidiol (CBD) in eyes of Zucker diabetic fatty rats

**DOI:** 10.1186/s42238-026-00421-z

**Published:** 2026-03-17

**Authors:** Barbara Takács, Anna Szilágyi, Panna Ötvös, Dániel Priksz, Mariann Bombicz, Adrienn Mónika Szabó, Zita Wachal, Rudolf Gesztelyi, Zoltán Szabó, Zoltán Szilvássy, Béla Juhász, Balázs Varga

**Affiliations:** 1 Department of Pharmacology and Pharmacotherapy, Faculty of Medicine, University of Debrecen, Nagyerdei Krt. 98., Debrecen, 4032 Hungary; 2 Doctoral School of Nutrition and Food Sciences, Nutrition Science Program, University of Debrecen, Debrecen, Hungary; 3 Department of Emergency Medicine, Faculty of Medicine, University of Debrecen, Debrecen, Hungary

**Keywords:** Cannabidiol (CBD), Retina, Ischemia–reperfusion, Electroretinography (ERG), Zucker Diabetic Fatty (ZDF) rat

## Abstract

**Background:**

Cannabidiol (CBD), main non-psychoactive ingredient of *Cannabis sativa* L. is known to have anti-ischemic, antidiabetic and neuroprotective effects. Ischemia–reperfusion injury of the retina is reportedly involved in deterioration of its function in diabetic retinopathy. The study was aimed to evaluate the in vivo potential retinoprotective role of CBD in type II diabetes mellitus.

**Methods:**

Zucker Diabetic Fatty (ZDF) rats were treated with CBD orally and electroretinographical analysis was carried out. In order to confirm the disease model and to assess other antidiabetic effects of CBD in ZDF rats, fasting blood glucose, oral glucose tolerance test, weight measurements and histology also took place.

**Results:**

Weight and glucose-related analyses supported our used diabetic animal model. CBD reduced diabetic weight gain without affecting glucose levels of ZDF rats, suggesting glucose-independent mechanisms of its retinal actions. CBD treatment selectively increased dark-adapted ERG amplitudes, while leaving light-adapted responses unaffected. It did not alter reduced amplitudes and prolonged implicit times of diabetic oscillatory potentials (OPs), but lowered variability of OP amplitudes and flicker peak intervals. A potential indirect mechanism for its effects beside glutamatergic inhibition is the ability of CBD to decrease thickening of diabetic retina.

**Conclusions:**

CBD exerted glucose-independent retinoprotective effects in ZDF rats by normalizing weight gain and preventing retinal thickening. While glycemic levels were unaffected, CBD selectively enhanced rod-mediated ERG amplitudes and reduced variability indicators of OPs and flickers.

**Supplementary Information:**

The online version contains supplementary material available at 10.1186/s42238-026-00421-z.

## Background

Cannabidiol (CBD) is one of the main components of *Cannabis sativa* L. beside the famous psychoactive ingredient, the tetrahydrocannabinol (THC), a closely related derivative (Fava et al. 2024). CBD acts on multiple cannabinoid receptors, including the two prominent CB1 and CB2 receptors, as well as the orphan G protein-coupled receptors (GPR) GPR55, GPR18, and GPR119 (Irving et al. 2017; Ye et al. 2019). Effects through CB1 and CB2 are generally mediated by Gi signal transduction cascade. However, CB1 is also able to couple to the counteractive Gs type G-protein, based on localization and/or activating ligand (Ye et al. 2019). And the co-localization of CB1/2 with GPR-type receptors (e.g., GPR55) likely complicates action-mechanisms further (Ye et al. 2019; Ramirez-Orozco et al. 2019; Papadogkonaki et al. 2019). Of note, CBD can also act on the transient receptor potential vanilloid (TRPV) and the peroxisome proliferator-activated receptor gamma (PPARγ), or modulate endocannabinoid signaling indirectly (Pisanti et al. 2017; Peng et al. 2022). Endocannabinoids themselves, such as arachidonyl-ethanolamine (anandamide, AEA) or 2-arachidonoyl-glycerol (2-AG), are phospholipid-derived ligands and enzymatically cleaved from the cell membrane (Papadogkonaki et al. 2019; Prospero-Garcia et al. 2019; Chen et al. 2005).

A complex consequence of these facts is that cannabinoid effects can be very diverse, and even opposing effects are conceivable depending on tissue specificity, dose, or ligand. The principal effects of CBD encompass anti-epileptic, anxiolytic, and anti-psychotic actions. In diabetes, CBD may improve outcomes by mitigating pancreatic inflammation, diabetic neuropathy, and contributing to metabolic homeostasis (O’Sullivan et al. 2023; Swenson 2025). Beyond these, CBD also shows promise for improving sleep quality, alongside possessing antiemetic and analgesic properties (O’Sullivan et al. 2023; Swenson 2025). Furthermore, CBD demonstrates notable organ-protective anti-ischemic effects, as have been exhibited in models of cerebral (Xu et al. 2023), cardiac (Naya et al. 2024), and renal ischemia (Fouad et al. 2012).

In the retina, CB1, CB2, GPR55, and GPR18 have been detected in different mammals (Straiker et al. 1999; Lopez et al. 2011; Bouskila et al. 2013; Cherif et al. 2015; MacIntyre et al. 2014). However, evidence for GPR119 remains indirect (Sloan et al. 2023). Endocannabinoids in the retina may regulate retinal development (Cherif et al. 2015; Silva et al. 2018), neuroprotection (Slusar et al. 2013; Nucci et al. 2007; Krishnan and Chatterjee 2014), and neurotransmission (Schwitzer et al. 2016), particularly the electrophysiology of scotopic vision (Bouskila et al. 2016).

CBD, as the main exocannabinoid, also exhibits notable retinal activity on its own. For instance, El-Remessy et al. have proved its role in counterbalancing NMDA-excitotoxicity in glaucoma through innate antioxidant actions (El-Remessy et al. 2003). They further demonstrated CBD´s capacity to mitigate type I diabetic retinal pathology by reducing vascular permeability and cell death (El-Remessy et al. 2006). The underlying mechanisms involve alleviating oxidative stress, inhibiting p38 MAPK signaling, and suppressing the levels of tumor necrosis factor-α, vascular endothelial growth factor, and intercellular adhesion molecule-1 (El-Remessy et al. 2003, 2006). Other researchers indicated that CBD exerts anti-inflammatory effects in the retina by enhancing adenosine signaling, specifically through the inhibition of adenosine uptake (Liou et al. 2008). More recently, Conti et al. revealed CBD´s neuroprotective effects in a model of AMPA-induced retinal damage (Conti et al. 2025). However, the potential in vivo retinoprotective role of CBD in type II diabetes mellitus remains undetermined. To address this, the present study aimed to evaluate this role in Zucker Diabetic Fatty (ZDF) rats.

## Materials and methods

### Animals and groups

**Fig. 1:**
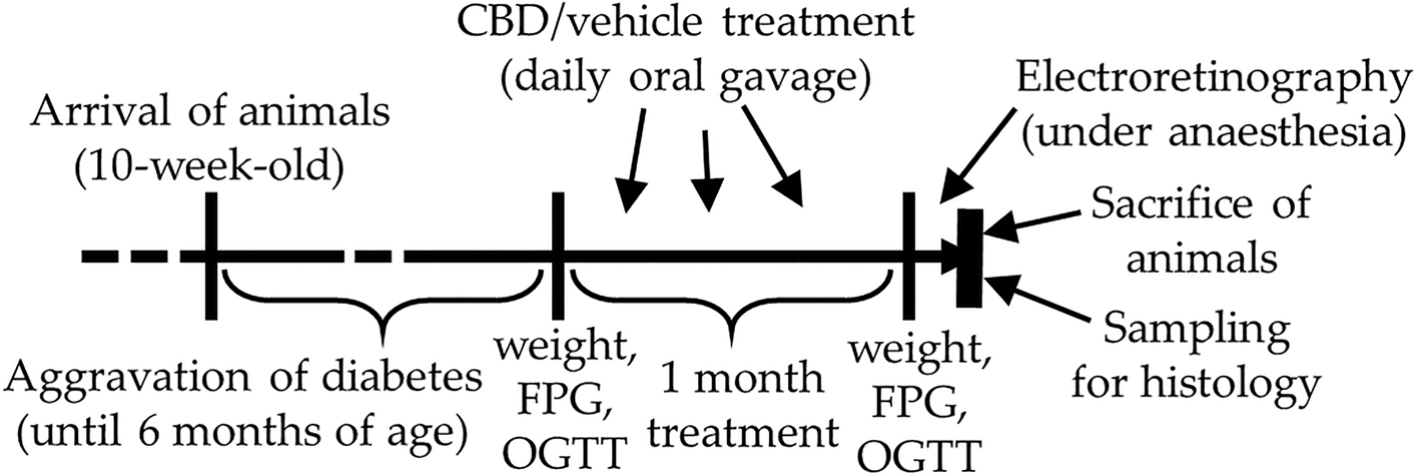
Flowchart of the research

Zucker Diabetic Fatty (ZDF) rats and their control, Lean rats, were bought from international animal retailer (Charles River Laboratories International, Inc. (Wilmington, MA, USA)) at the age of 10 weeks. ZDF rats, used as Type 2 diabetes model animals, develop glucose intolerance and hyperinsulinemia, hyperglycemia and its consequences due to their leptin-resistance caused by mutation (Al-Awar et al. 2016). The animals were kept in our animal house with ad libitum water and standard rodent chow until 6 months of age after which 1 month of treatment commenced. Three groups were formed (*n*=6): ZDF control, CBD-treated ZDF rats and Lean. The number of animals was calculated to align with current regulatory requirements to minimize animal use in research, although the small sample size might be considered as a limitation of the study. ZDF rats were assigned randomly to CBD-treated or vehicle-treated groups. Orally gavaged through a feeding tube, vehicle treatment consisted of sunflower oil, while CBD was dissolved in the same oil in 60 mg/kg/day dosage. The used dose was based on human oral acceptable daily intake (ADI) dose (10 mg/kg/day) established by Food Standards Agency (2023) and on rat doses used in other studies (Singh et al. 2023; Hlozek et al. 2017). Flowchart of the research is shown in Fig. 1.

All treatments and protocols received ethical clearance by the Laboratory Animal Research Committee of the University of Debrecen (9/2022/DEMÁB) and were conducted in accordance with the 3R principles (replacement, reduction and refinement) and ARVO Statement for the Use of Animals in Ophthalmic and Vision Research. The animals received humane care corresponding to the ‘Principles of Laboratory Animal Care’ by EU Directive 2010/63/EU.

### Fasting blood glucose

To validate the diabetic animal model, glucose related measurements were carried out as detailed in this and the following chapter. The animals underwent an overnight fasting and on the following morning fasting blood glucose was measured. For recording Accu-Check glucose meter (Roche Diagnostics, Mannheim, Germany) was used; blood was collected from the tail vein of rats (Wachal et al. 2020).

### Oral Glucose Tolerance Test (OGTT)

Oral glucose tolerance test was carried out by a formerly used protocol (Wachal et al. 2021). Briefly, after measuring their fasting blood glucose, animals were gavaged 2 g/bwkg of glucose through a feeding tube. Blood glucose levels were measured from tail vein repeatedly at 15, 30, 60, 90 and 120 minutes using Accu-Check glucose meter (Roche Diagnostics, Mannheim, Germany) and a time-glucose level curve was constructed from the results.

### Electroretinography

Our electroretinography (ERG) method, as also described previously (Takacs et al. 2024), is an electrophysiological measurement which makes it possible to analyze *in vivo* the electrical functioning of the retina upon light stimulus. The used protocol is based on the current standard electroretinography protocol recommended by International Society for Clinical Electrophysiology of Vision (ISCEV) for both clinical application and research purposes (Robson et al. 2022). A Ganzfeld-type flash lamp is utilized for stimulus generation and a Hand-held Multi-species ElectroRetinoGraph (HMsERG, OcuScience, Henderson, NV, USA) for data acquisition, both fixed securely in position inside a Faraday-cage along with a heated pad. The rat lies in a prone position on the heated pad (ATC 2000, WPI, Sarasota, FL, USA); ERG measurements are performed under general anesthesia (ketamine-xylazine; 100/10 mg/kg) and mydriasis (cyclopentolate, Humapent, Teva Ltd., Debrecen, Hungary). For conduction of retinal electrical activity the method utilizes 5 electrodes: two corneal contact lens electrodes (ERG-jet Contact Lens Electrode, Fabrinal SA, Switzerland) with conductive gel and two reference needle electrodes, the latter being inserted into the cheek of the animal; the system also includes a general earth needle electrode inserted into the midline of the animal.

Measurements are first carried out after a 20-minute dark adaptation, later with standard (30 cd·s/m^2^) background light adaptation. The HMsERG is capable of registering electrical activity of both eyes simultaneously with a bandpass filter (1 to 300 Hz width) and with time-integrated luminance (strength) of a single flash ranging from −2.5 to 1 log cd·s/m^2^ (10, 100, 300, 1000, 3000, 10000, 25000 mcd·s/m^2^). Electroretinograms (a-waves, b-waves, their implicit times, oscillatory potentials (OP) and flickers) were analyzed with ERGView software (version 4.380, OcuScience, Henderson, NV, USA).

The commonly analyzed a- and b-waves were identified, measured and their implicit times were calculated by the software automatically, while in case of OPs and flickers, the following two methods were used within the same software. To make OPs visible, after acquisition of the electroretinogram a bandpass filter of 100 to 300 Hz was applied. Waveform morphology, amplitude and implicit time of OPs of 3000 mcd·s/m^2^ stimuli were evaluated as regular (Wachtmeister 1998). The maximal amplitude of oscillatory potentials was measured from the baseline of 0 µV selecting the highest peak and the lowest trough (a negative number), absolute values of which were then summed up together. The implicit time of OPs were measured between the flash stimulus and the highest peak. Results were averaged from 4-4 OP-measurements from each eye of all animals in a group. Variabilities for OP amplitudes were calculated by GraphPad Prism software as Coefficient of Variation, dividing the Standard Deviation of the OP amplitude data-points with their mean. Flicker method was carried out after the light-adapted recordings (with the same backlight) with 30.30 Hz, 3000 mcd·s/m^2^ light stimuli. Intervals between two consecutive highest peaks following the light stimulus were analyzed. Results were averaged from 4-4 flicker measurements of animals of each group. Variabilities of flicker peak intervals were calculated similarly to OP amplitude variability by GraphPad Prism software.

### Histology

For histological analysis, enucleated eyes of the animals were sectioned after embedding them into Histowax (Histolab Products AB, Gothenburg, Sweden) as discussed in details before (Takacs et al. 2024). Briefly, right after enucleation, samples were fixated in paraformaldehyde solution (PFA, pH 7.4, 4% in phosphate buffer: 10 g paraformaldehyde, 50 µL 10 N NaOH, 25 mL 10× PBS, 200 ml ddH2O), which was later washed out and the tissue was dehydrated in ascending alcohol and xylene. Four µm thick sections were then cut from the tissue blocks with a microtome. After deparaffinization and rehydration, sections were stained with hematoxylin and eosin (Sigma-Aldrich-Merck KGaA, Darmstadt, Germany). Sections (*n*=10/animal) were analyzed with a Nikon Eclipse 80i microscope through a 40× objective (Nikon Plan Fluor 40×/0.75 DIC M/N2 ∞/0.17 WD 0.66) with a DS-Fi3 Microscope Camera attached. Thickness of the layers were measured with the software of the microscope (Nikon NIS-Elements BR, Ver5.41.00).

### Statistical analysis

Statistical analyses were done in GraphPad Prism software (version 8.0, GraphPad Software Inc., La Jolla, CA, USA) as discussed before (Takacs et al. 2024). Briefly, after Shapiro-Wilk normality test for determination of distribution of data points, either one-way analysis of variance (one way ANOVA), or non-parametric Kruskal-Wallis test, or two-way analysis of variance (two-way ANOVA) was used. Comparisons were regarded significant in case of a lower than 0.05 probability value. Various signs (*, #) were used to mark significance in different comparisons (e.g. * to **** for *p* < 0.05, *p* < 0.01, *p* < 0.001, and *p* < 0.0001, respectively; and similarly with #). Data were presented as mean and standard error of the mean (SEM).

## Results

**Fig. 2:**
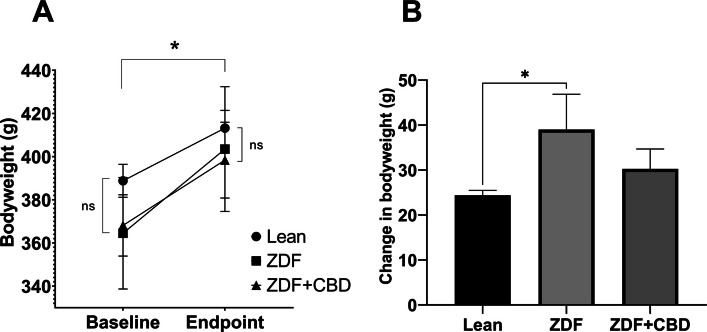
Bodyweight changes in the different animal groups. **A** Baseline and endpoint bodyweight values; results are group means ± SEM, ns = no significant difference in any possible comparisons between groups in a given time point, * = *p* < 0.05 in any comparisons between group values within the same group in different time points. **B** Change in bodyweight in the different animal groups. Results are group means + SEM, * marks statistically significant comparison (*p* < 0.05)

Results of bodyweight measurements are seen in Fig. 2, according to which baseline mean values (±SEM) of the groups were measured to be 388.8 ± 7.615, 364.5 ± 25.825 and 368.125 ± 14.181 grams, for Lean, ZDF and ZDF+CBD groups, respectively, while endpoint values were 413.2 ± 8.208, 403.5 ± 28.895 and 398.375 ± 17.557 grams, in the same order. There were significant differences between baseline and endpoint weights in all groups (effect of time factor), which is shown as the horizontal pairwise comparison in Fig. 2A, but there were no differences between the groups in any time points (effect of group factor), which is shown as the vertical pairwise comparisons on both sides of Fig. 2A. Opposed to this, the change in bodyweight (the slope of the lines in Fig. 2A) were significantly different between groups: untreated ZDF animals gained significantly more weight than the Lean group (Fig. 2B). Values of weight-change in Lean, ZDF, ZDF+CBD group were 24.40 ± 1.067, 39.00 ± 7.853 and 30.25 ± 4.427 grams, respectively.

**Fig. 3:**
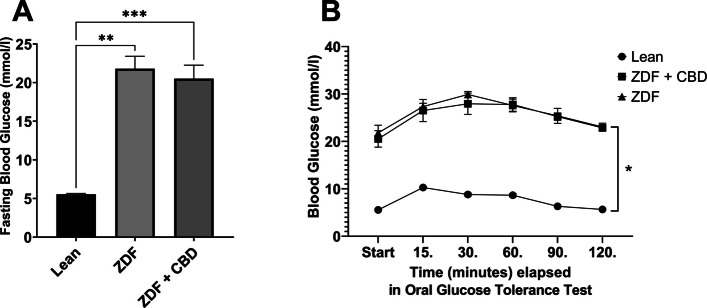
Glucose measurements at the end of the study. **A** fasting blood glucose values; plotted results are group means + SEM, ** = *p* < 0.01; *** = *p* < 0.001. **B** results of oral glucose tolerance test (OGTT); plotted results are shown as group means ± SEM (error bars shorter than the size of the symbols are not visible); * = *p* < 0.05 in comparison of Lean group with any ZDF group in any time points

Endpoint glucose measurements are shown in Fig. 3. Fasting blood glucose values were measured to be 5.550 ± 0.1078, 21.80 ± 1.613 and 20.53 ± 1.731 mmol/l for Lean, ZDF and ZDF+CBD groups, respectively (Fig. 3A). According to the OGTT measurements glucose values of the Lean group in any time points significantly differed from the other two groups, but there were no significant differences between treated and untreated ZDF groups in any time points (Fig. 3B).

**Fig. 4:**
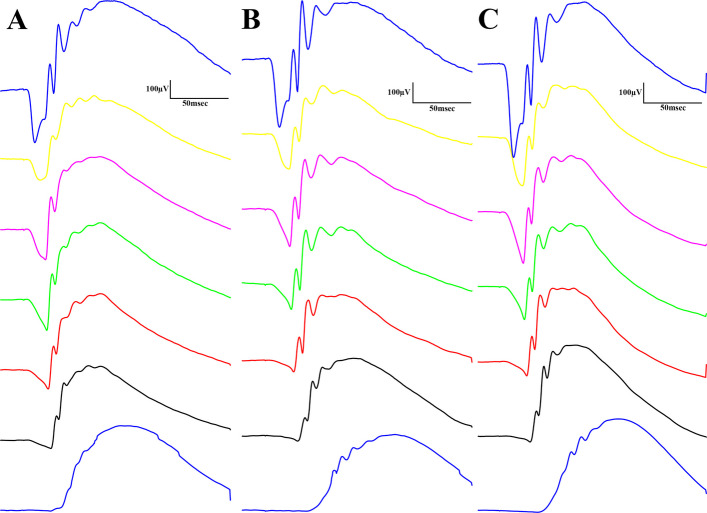
Dark-adapted electroretinographical waveforms elicited by flash light series (from the bottom to the top: 10, 100, 300, 1000, 3000, 10000, 25000 mcd·s/m^2^). **A** Lean group; **B** ZDF group; **C** ZDF + CBD group

**Fig. 5:**
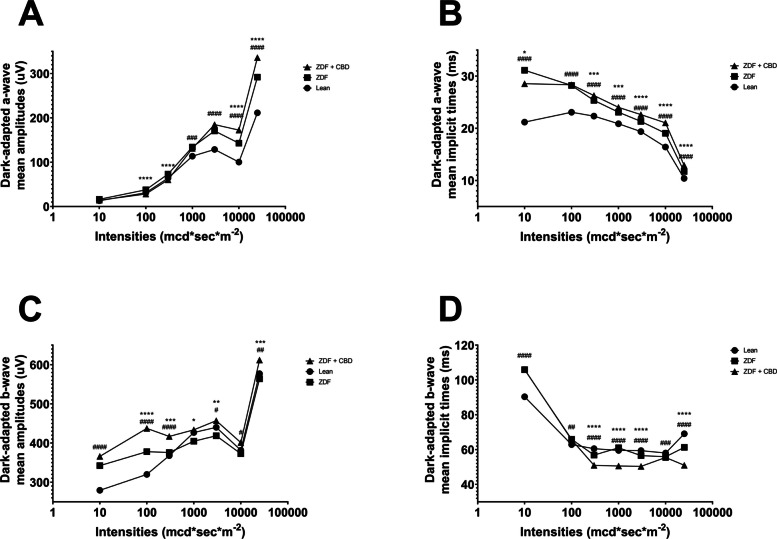
Wave amplitudes and implicit times plotted as a function of increasing dark-adapted flashlight strength (mcd·s/m^2^). **A** Dark-adapted a-wave mean amplitudes (µV); **B** Dark-adapted a-wave mean implicit times (msec); **C** Dark-adapted b-wave mean amplitudes (µV); **D** Dark-adapted b-wave mean implicit times (msec). Dots represent Lean group, squares ZDF group, triangles ZDF + CBD group. All values are presented as group means. Statistically significant comparisons are marked with * in case of ZDF vs ZDF + CBD comparisons, and # in case of Lean vs ZDF + CBD comparisons. The number of markers represents the statistical significance of the comparison * or # *p* < 0.05; ** or ## *p* < 0.01; *** or ### *p* < 0.001; **** or #### *p* < 0.0001

**Fig. 6:**
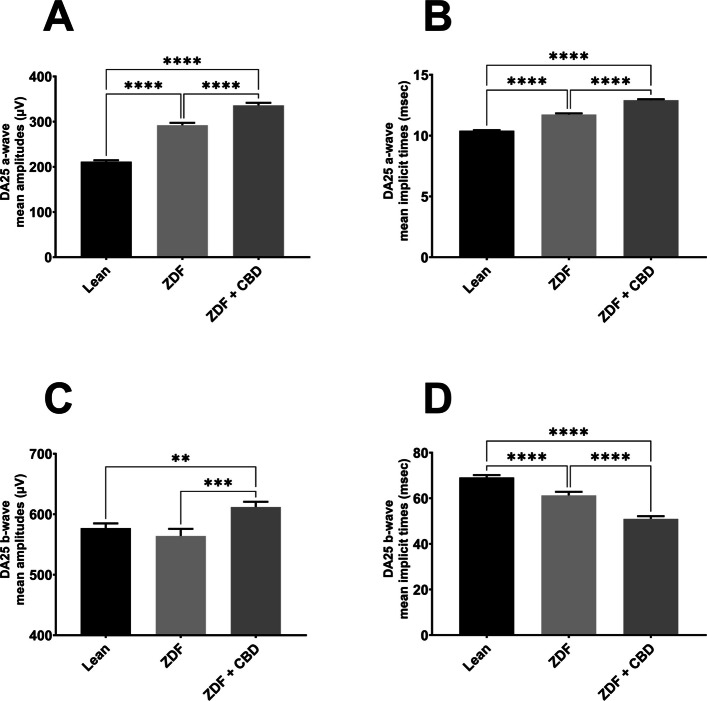
Dark-adapted ERG measurements with 25000 mcd·s/m^2^ flash light (DA25). **A** mean a-wave amplitudes of the different groups (µV); **B** mean a-wave implicit times (msec); **C** mean b-wave amplitudes (µV); **D** mean b-wave implicit times (msec). All results are shown as group means + SEM. ** = *p* < 0.01; *** = *p* < 0.001 and **** = *p* < 0.0001

Electroretinography results are shown in the several following figures as described below. Dark-adapted wave results are shown in Figs. 4, 5 and 6: representative waveforms are shown in Fig. 4, while mean amplitude and implicit time values are shown in Fig. 5, most important comparisons are detailed in Fig. 6.

According to dark-adapted ERG measurements a-wave as well as b-wave amplitudes were the highest in ZDF + CBD group on most stimulus strengths. Exact values are shown in Table 1. This was not the case with the implicit times, where we observed longer a-wave and shorter b-wave implicit times for ZDF + CBD group in most stimulus strengths. In particular, under DA 25 a-waves’ amplitude and their implicit times were significantly higher in the two ZDF groups compared to Lean, while ZDF + CBD group values were the highest significantly differing from untreated ZDF group as well. B-waves’ amplitude was significantly higher in ZDF + CBD group compared to Lean, while significantly lower in ZDF compared to ZDF + CBD group. Implicit times for b-waves were significantly the shortest in ZDF + CBD group compared to both Lean and ZDF groups.

**Table 1:** Dark-adapted ERG Values

			Lean	ZDF	ZDF + CBD
DA 0.01	a-wave	mean amplitudes (µV)	13.07 ± 0.6142	16.36 ± 0.9842	14.25 ± 0.8329
		mean implicit times (msec)	21.18 ± 0.4278	31.12 ± 0.7486	28.55 ± 0.7456
	b-wave	mean amplitudes (µV)	279.3 ± 5.829	342.3 ± 6.992	366 ± 7.053
		mean implicit times (msec)	90.36 ± 0.6108	105.9 ± 0.9913	106 ± 0.8041
DA 0.1	a-wave	mean amplitudes (µV)	31.01 ± 0.9771	37.76 ± 1.272	28.3 ± 1.024
		mean implicit times (msec)	23.07 ± 0.2514	28.21 ± 0.2828	28.35 ± 0.3271
	b-wave	mean amplitudes (µV)	319.8 ± 5.449	378 ± 7.494	437.4 ± 6.334
		mean implicit times (msec)	62.83 ± 0.4296	65.89 ± 0.9251	65.62 ± 0.7027
DA 0.3	a-wave	mean amplitudes (µV)	64.11 ± 1.71	73.06 ± 2.35	60.32 ± 1.674
		mean implicit times (msec)	22.34 ± 0.123	25.35 ± 0.1709	26.28 ± 0.1468
	b-wave	mean amplitudes (µV)	367.8 ± 5.873	375.8 ± 7.331	416.8 ± 6.181
		mean implicit times (msec)	60.52 ± 0.4237	56.82 ± 0.9392	50.95 ± 0.8038
DA 1	a-wave	mean amplitudes (µV)	113.6 ± 2.391	134.1 ± 3.195	131.1 ± 2.855
		mean implicit times (msec)	20.87 ± 0.08232	23.09 ± 0.1399	24.01 ± 0.1328
	b-wave	mean amplitudes (µV)	426.9 ± 6.349	404.6 ± 8.367	433.7 ± 7.251
		mean implicit times (msec)	59.49 ± 0.5341	61.01 ± 0.8563	50.57 ± 0.7761
DA 3	a-wave	mean amplitudes (µV)	128.8 ± 2.211	170.6 ± 4.116	184.9 ± 3.703
		mean implicit times (msec)	19.37 ± 0.09953	21.32 ± 0.1452	22.62 ± 0.1234
	b-wave	mean amplitudes (µV)	440.0 ± 6.090	418.6 ± 8.938	457.1 ± 7.258
		mean implicit times (msec)	59.39 ± 0.5610	56.55 ± 0.8108	50.35 ± 0.6789
DA 10	a-wave	mean amplitudes (µV)	100.3 ± 2.047	143 ± 5.328	172.4 ± 3.709
		mean implicit times (msec)	16.41 ± 0.1288	19.05 ± 0.2104	21.05 ± 0.1782
	b-wave	mean amplitudes (µV)	381.6 ± 5.623	372.8 ± 9.371	401.2 ± 6.95
		mean implicit times (msec)	57.99 ± 0.6299	55.87 ± 0.7553	55.46 ± 0.4867
DA 25	a-wave	mean amplitudes (µV)	211.5 ± 2.968	292.0 ± 5.368	336.1 ± 5.676
		mean implicit times (msec)	10.40 ± 0.04428	11.73 ± 0.09626	12.91 ± 0.08102
	b-wave	mean amplitudes (µV)	577.1 ± 7.689	564.1 ± 11.74	612.0 ± 8.722
		mean implicit times (msec)	69.10 ± 1.066	61.28 ± 1.559	50.99 ± 1.169

**Fig. 7:**
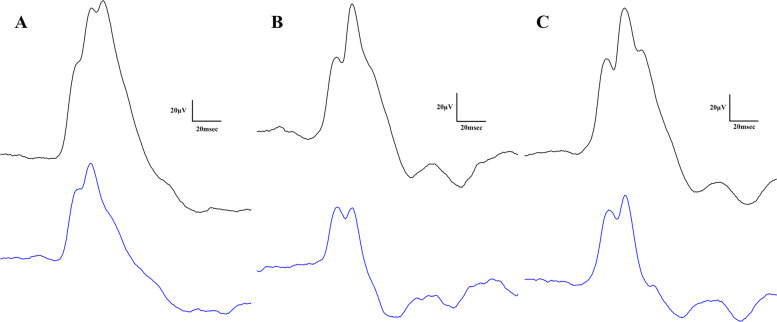
Representative light-adapted electroretinographical waveforms (blue line for 3000 and black line for 10000 mcd·s/m^2^ light stimuli). **A** Lean, **B** ZDF, **C** ZDF + CBD group

**Fig. 8:**
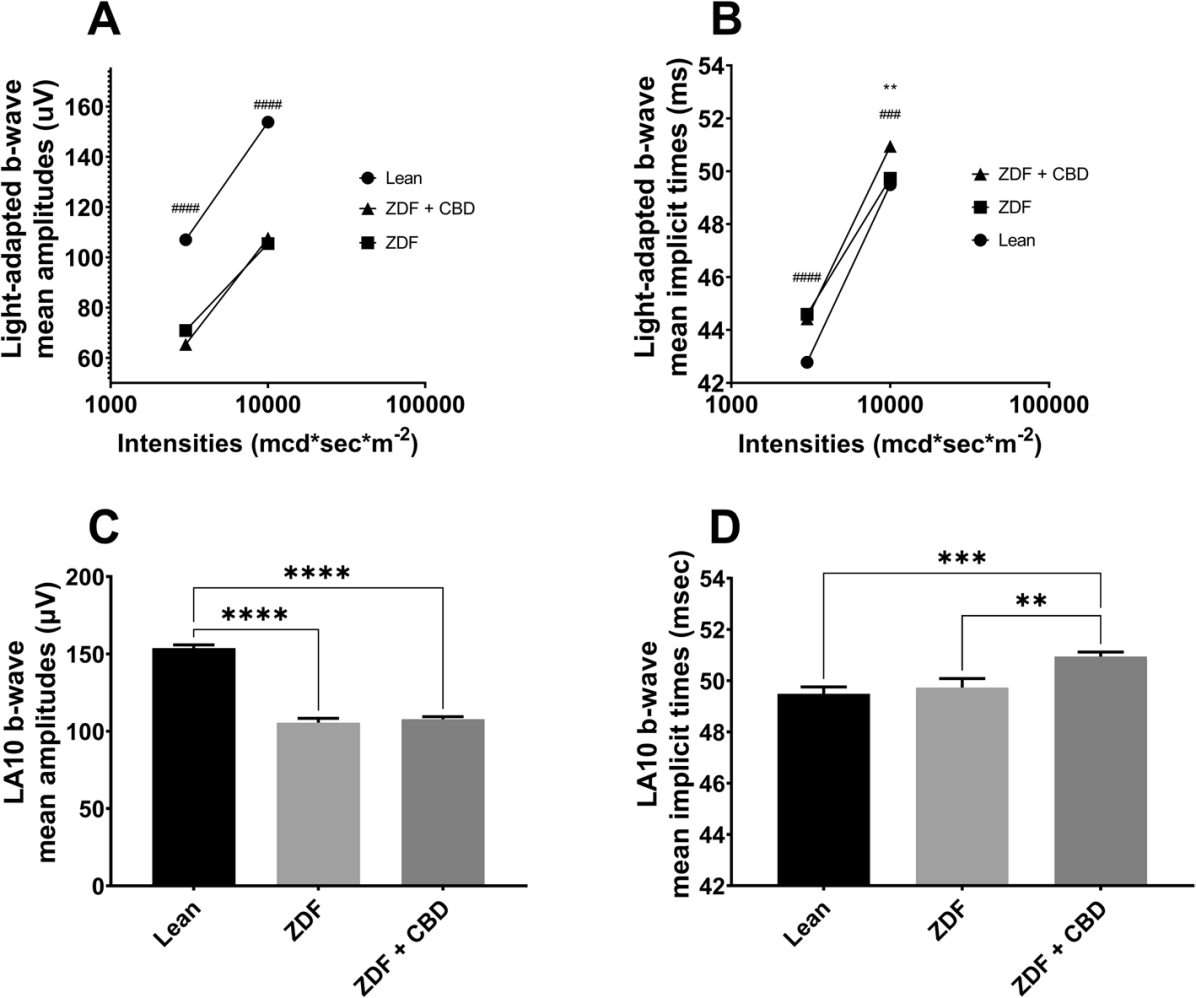
Results of light-adapted ERG measurements. **A** Light-adapted b-wave mean amplitudes (µV), **B** Light-adapted b-wave mean implicit times (msec) both plotted as a function of increasing light-adapted flash light strengths (mcd·s/m^2^). Dots represent Lean group, squares ZDF group, triangles ZDF + CBD group. All values are presented as group means. Statistically significant comparisons are marked with * in case of ZDF vs ZDF + CBD comparisons, and # in case of Lean vs ZDF + CBD comparisons. The number of markers represents the statistical significance of the comparison ** *p* < 0.01; ### *p* < 0.001 and #### *p* < 0.0001. **C** and **D** Light-adapted ERG measurements with light-adapted 10000 mcd·s/m^2^ flash light (LA10). **C** mean b-wave amplitudes (µV); **D** mean b-wave implicit times (msec). All results are shown as group means + SEM. ** = *p* < 0.01, *** = *p* < 0.001, **** = *p* < 0.0001

Light-adapted ERG results are shown in Figs. 7 and 8: representative waveforms are shown in Fig. 7, while numeric results are shown in Fig. 8.

Regarding light-adapted ERG measurements, we found no differences between amplitudes of treated and non-treated ZDF groups. Amplitudes of ZDF + CBD group were significantly lower than in Lean group under both lightings, while implicit times were significantly longer in this group compared to Lean group in case of both stimulus strengths. Exact values are shown in Table 2.

**Table 2:** Light-adapted ERG values

			Lean	ZDF	ZDF + CBD
LA3	b-wave	mean amplitudes (µV)	107.0 ± 1.504	70.92 ± 2.247	65.37 ± 1.318
		mean implicit times (msec)	42.78 ± 0.1409	44.59 ± 0.3230	44.42 ± 0.3030
LA10	b-wave	mean amplitudes (µV)	153.8 ± 2.047	105.5 ± 2.281	107.7 ± 1.676
		mean implicit times (msec)	49.49 ± 0.2698	49.73 ± 0.3537	50.94 ± 0.1755

**Fig. 9:**
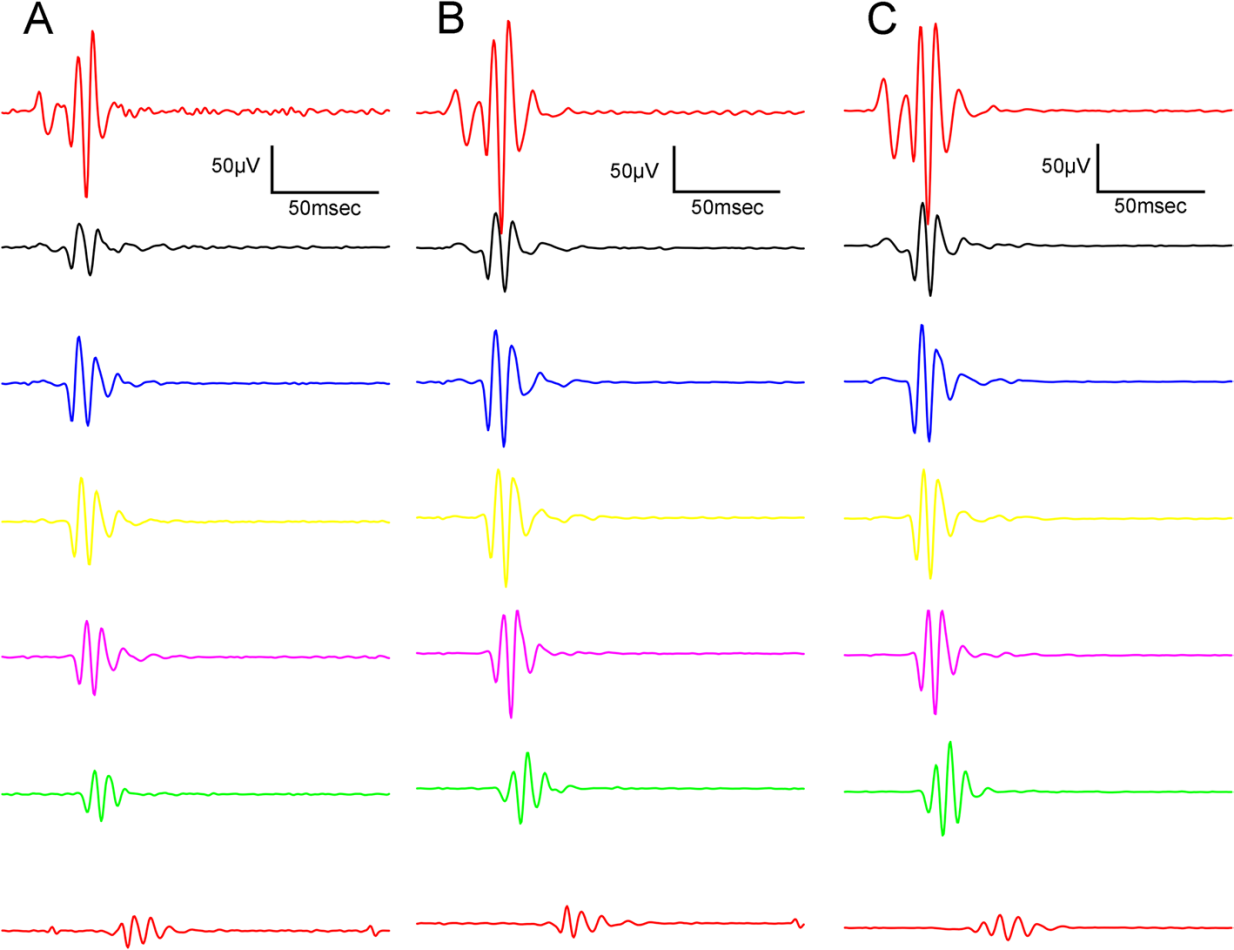
Dark-adapted oscillatory potential (OP) waveforms elicited by flash light series (from the bottom to the top: 10, 100, 300, 1000, 3000, 10000, 25000 mcd·s/m^2^). **A** Lean group; **B** ZDF group; **C** ZDF + CBD group

**Fig. 10:**
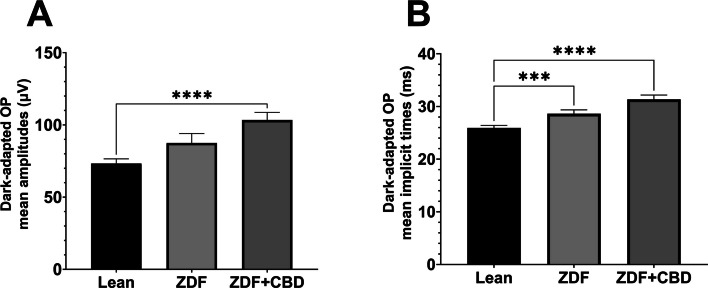
Mean amplitudes (**A**) and mean implicit times (**B**) of dark-adapted oscillatory potentials (OPs) of 3000 mcd·s/m^2^ stimuli. All results are shown as group means + SEM. *** = *p* < 0.001 and **** = *p* < 0.0001

**Fig. 11:**
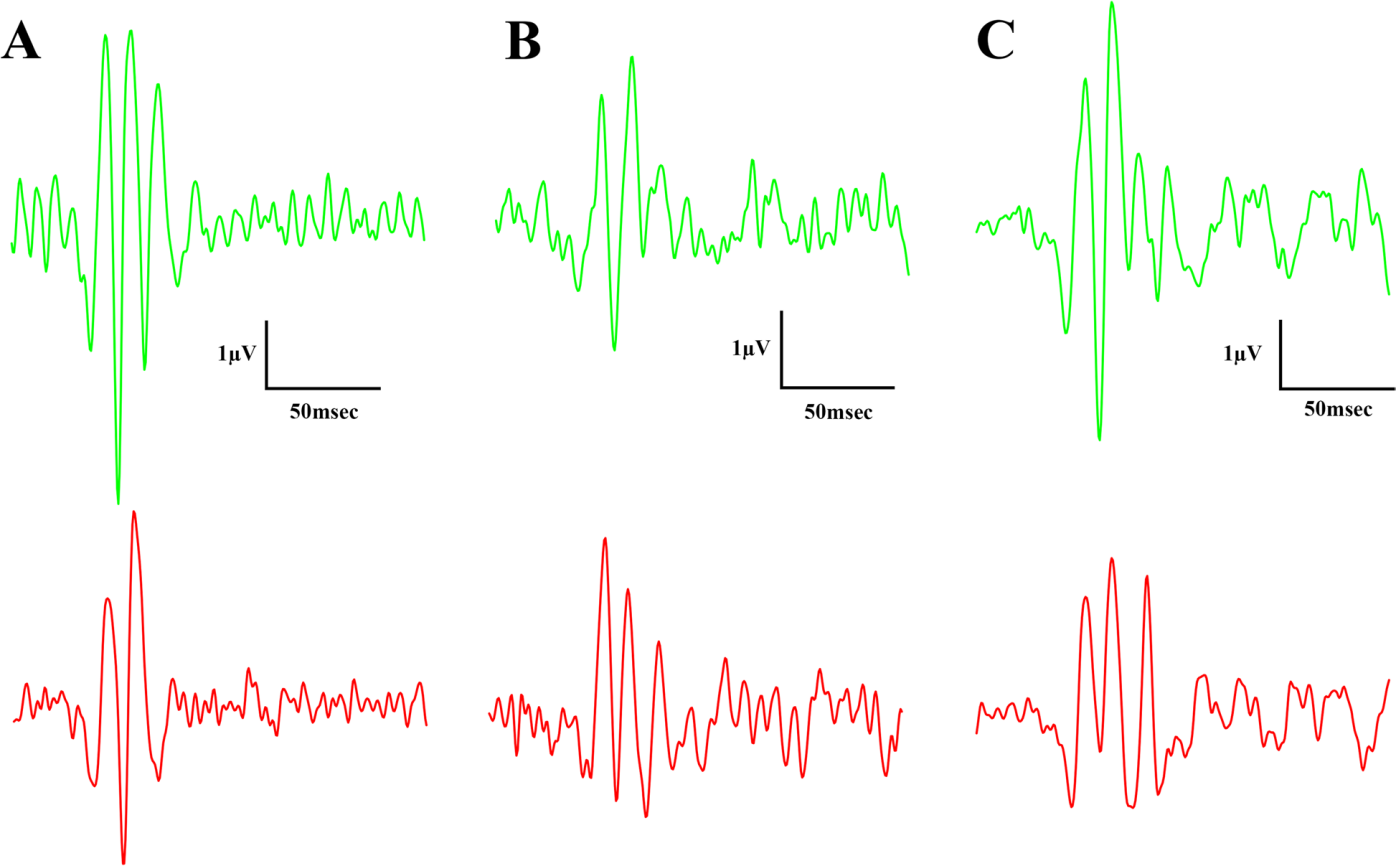
Light-adapted oscillatory potential (OP) waveforms elicited by flash light strengths (from the bottom to the top) 3000 and 10000 mcd·s/m^2^. **A** Lean group; **B** ZDF group; **C** ZDF + CBD group

**Fig. 12:**
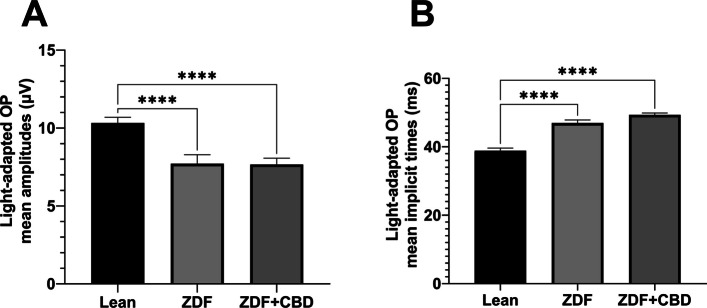
Mean amplitudes (**A**) and mean implicit times (**B**) of light-adapted oscillatory potentials (OPs) of 3000 mcd·s/m^2^ stimuli. All results are shown as group means + SEM. **** = *p* < 0.0001

**Fig. 13:**
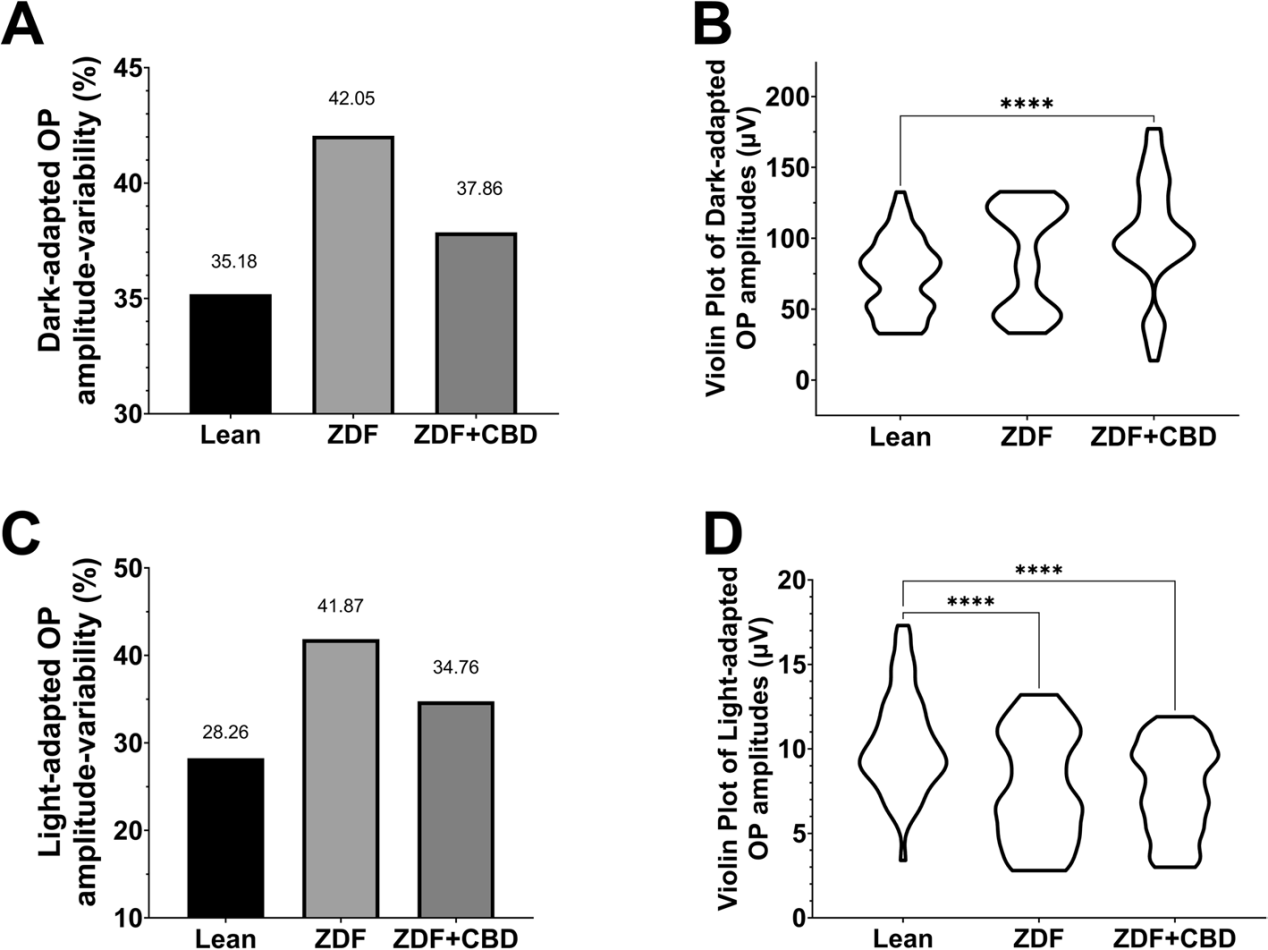
Variability of Oscillatory Potentials (OPs) of 3000 mcd·s/m^2^ stimuli. **A** Variability of Dark-adapted OP amplitudes and **B** Violin Plot of Dark-adapted OP amplitudes. **C** Variability of Light-adapted OP amplitudes and **D** Violin Plot of Light-adapted OP amplitudes. Variabilities are in percentages (%) while the individual measurements are plotted based on the distribution according to amplitudes in µV units. Due to the nature of the calculation used for variabilities, no standard deviations (SD) or standard errors of the means (SEM) could be indicated in variability plots, hence no statistical correlations could be calculated between groups

Oscillatory potentials were also recorded during the experiment: Figs. 9 and 10 show dark-adapted while Figs. 11 and 12 light-adapted oscillatory potential data, furthermore Fig. 13 shows variability of oscillatory potentials.

The waveform morphology of dark-adapted oscillatory potentials of animals in both ZDF animal groups resembled that of untreated lean animals (Fig. 9); there were no extra or missing peaks and the course of the curves also showed great similarity. The measured mean amplitudes were, nevertheless, significantly higher in the ZDF+CBD group compared to the lean group (103.4±5.278 vs 73.26±3.222 µV, respectively; *p*<0.0001). The untreated ZDF group did not differ from neither of the other two groups (87.51±6.505 µV). There was a similar trend in case of the implicit times of the oscillatory potentials, where we recorded significant longer implicit times for the ZDF + CBD group compared to Lean group (31.34±0.8318 vs 25.93±0.4688 msec, respectively; *p*<0.0001). Here the untreated ZDF group also differed significantly from the Lean group (28.62±0.7310 vs 25.93±0.4688 msec, respectively; *p*<0.001).

There were slight differences between the waveform morphology of the light-adapted oscillatory potentials of ZDF animals and Lean animals. At the lower light strength an extra wave was recorded in ZDF animals beyond the two peaks seen in case of Lean animals, while at the higher light strength a third peak was recognizable even in case of Lean animals, however, this peak was much smaller in case of ZDF animals. Regarding the maximal amplitude and the time to reach this maximal amplitude measurements showed significant differences between Lean and ZDF groups: mean amplitudes of Lean light-adapted oscillatory potentials were significantly higher than in any ZDF groups (10.33±0.3647 vs 7.719±0.5713 and 7.666±0.4017 µV, for Lean vs ZDF and ZDF + CBD groups respectively; *p*<0.0001), while implicit times were longer in ZDF animals compared to Lean (38.85±0.7678 vs 46.94±0.9039 and 49.33±0.5593 msec, for Lean vs ZDF and ZDF + CBD groups respectively; *p*<0.0001).

Variabilities for the measured Oscillatory Potentials were calculated by GraphPad Prism software as Coefficient of Variation, dividing the Standard Deviation of the OP data points with their mean (Fig. 13A and C). Variability tells us about the scatter of OPs, which is also graphically shown as a violin plot in Fig. 13B and D. Since neither Standard Deviation (SD) nor Standard Error of the Mean (SEM) could be associated to the bars of the graphs in Fig. 13, due to the nature of the calculation of Coefficient of Variation, no statistical analyses could be carried out on the data, however, the trend is clearly visible: in case of the variability of all OP amplitudes measured in the group of ZDF + CBD treated animals, percentage of variability resembles more to that of Lean group, while ZDF group variability is much higher both in case of dark-adapted and Light-adapted OP-measurements. A similar trend can be seen in the violin plots (Fig. 13B and D) where the scatter of the individual OPs are illustrated: the most OP data points of ZDF + CBD animals lie in the same range as in case of Lean animals, while non-treated ZDF data points show a dumbbell-shaped distribution.

**Fig. 14:**
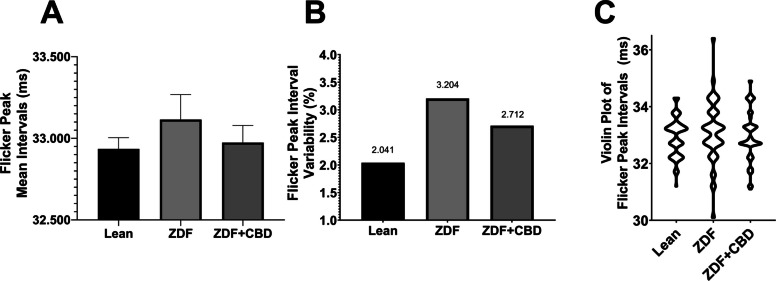
Results of flicker measurements of 3000 mcd·s/m^2^ stimuli. **A** Mean intervals of flicker peaks (msec). **B** Variability of flicker peak intervals (%). **C** Violin plot of flicker peak intervals (msec). Due to the nature of the calculation used for variability, no standard deviations (SD) or standard errors of the means (SEM) could be indicated in variability plot, hence no statistical correlations could be calculated between groups

Flicker measurement results are shown in Fig. 14.

According to flicker measurements there were some minor differences between the mean intervals of flicker peaks: mean interval of Lean group turned out to be 32.94±0.06862 msec, that of ZDF + CBD group was measured to be 32.97±0.1054 msec while in case of untreated ZDF group this interval was 33.11±0.1532 msec, however, the differences did not reach the level of statistical significance. The variability of the flicker peak intervals were the highest in the ZDF group, and the range was also the greatest in this group, while results of the ZDF + CBD group resembled more, both in case of variability and distribution, to the results of Lean animals. Range of individual interval values were 3.1 (31.2 - 34.3) msec in Lean group, 3.8 (31.1 - 34.9) msec in ZDF + CBD group and the most scattered 6.3 (30.1 - 36.4) msec in ZDF group as depicted in Fig. 14C.

**Fig. 15:**
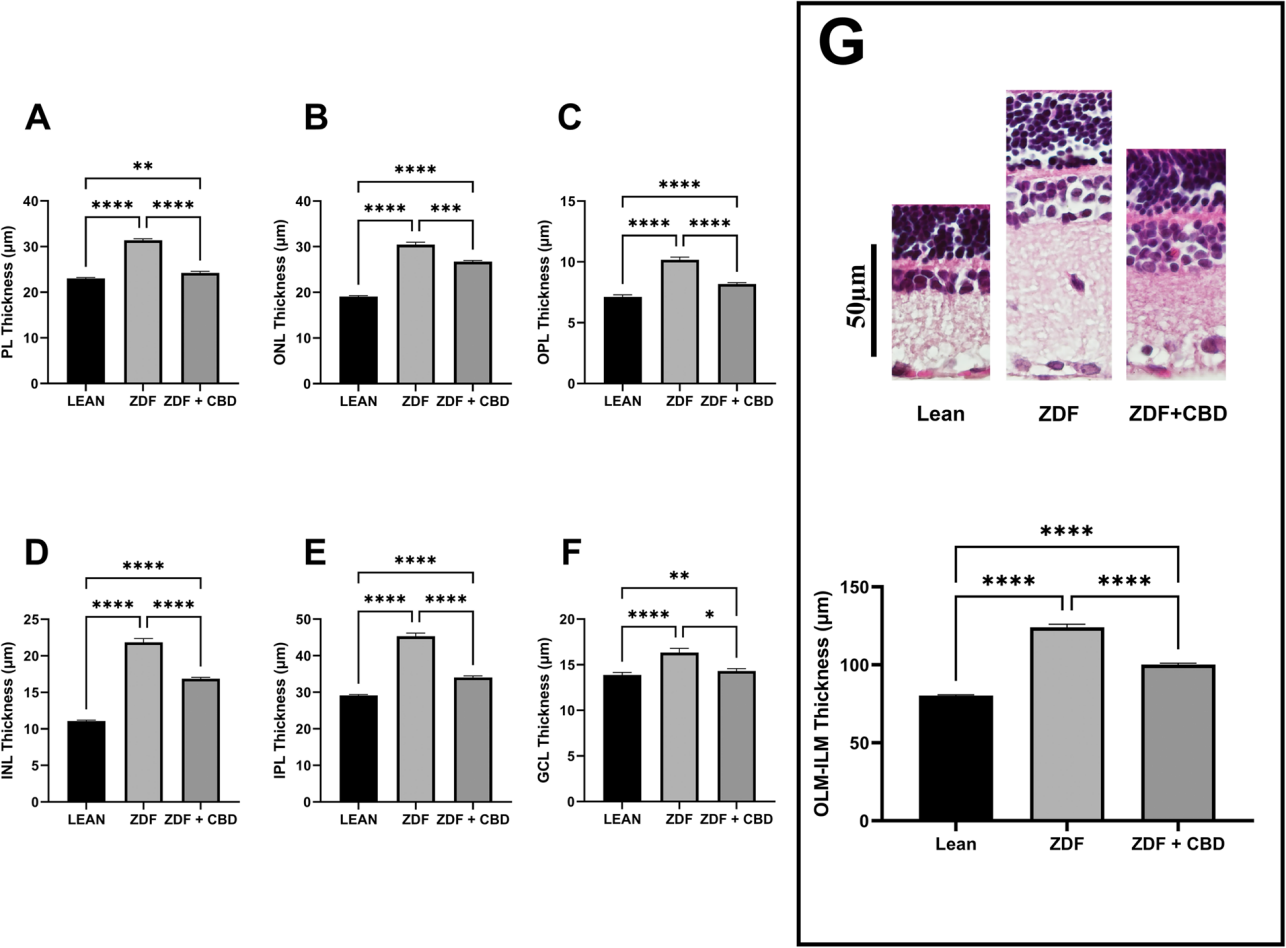
Histology Results – Mean thickness of the retinal layers across animal groups. **A** Photoreceptor Layer (PL), **B** Outer Nuclear Layer (ONL), **C** Outer Plexiform Layer (OPL), **D** Inner Nuclear Layer (INL), **E** Inner Plexiform Layer (IPL), and **F** Ganglion Cell Layer (GCL). **G** Representative histological sections of the different groups (from left to right): Lean, ZDF, ZDF + CBD and Graphs showing the overall thickness of retinal layers from OLM to ILM (outer to inner limiting membrane). Data are shown as group mean + SEM. * = *p* < 0.05, ** = *p* < 0.01, *** = *p* < 0.001, **** = *p* < 0.0001

Results of histological analysis are shown in Fig. 15.

According to histological analysis significant differences were measured between the thicknesses of the different layers of the retina in the different treatment groups: in every measurement layer thicknesses of ZDF animals were the highest, while those of ZDF+CBD group were significantly lower than ZDF, resembling Lean. As with all layers measured separately, in case of OLM-ILM thickness as well, ZDF group had significantly higher values compared to both Lean and ZDF+CBD groups. Individual values of bar graphs of all measurements are shown in a supplementary figure. Exact retinal thickness values of histological analysis are shown in Table 3.

**Table 3:** Histology – retinal thickness values

		Lean	ZDF	ZDF + CBD
Retinal thickness	photoreceptor layer (PL)	23.02 ± 0.2033	31.38 ± 0.3228	24.25 ± 0.3183
	outer nuclear layer (ONL)	19.07 ± 0.2085	30.44 ± 0.5596	26.72 ± 0.2713
	outer plexiform layer (OPL)	7.114 ± 0.1743	10.17 ± 0.2192	8.187 ± 0.1111
	inner nuclear layer (INL)	11.08 ± 0.1195	21.83 ± 0.5268	16.86 ± 0.2061
	inner plexiform layer (IPL)	29.16 ± 0.2490	45.32 ± 0.8474	34.07 ± 0.4156
	ganglion cell layer (GCL)	13.88 ± 0.2741	16.32 ± 0.4698	14.31 ± 0.2566
	outer to inner limiting membrane (OLM-ILM)	80.31 ± 0.5422	124.1 ± 2.033	100.2 ± 0.8944

## Discussion

In our study Zucker Diabetic Fatty (ZDF) rats were treated with Cannabidiol (CBD) in order to evaluate in vivo potential retinoprotective role of CBD in type II diabetes mellitus.

### Effects on bodyweight and glucose levels

In line with scientific literature ZDF animals did not become obese, but instead remained on a lower bodyweight (Srinivasan and Ramarao 2007). However, untreated ZDF group exhibited a faster growth rate compared to Lean animals (Fig. 2), which was likely attributable to diabetic metabolic changes (Schuster and Duvuuri 2002). CBD treatment normalized weight gain to levels comparable with the Lean group. Given the absence of distress signs, this normalization suggests a systemic metabolic effect of CBD, extending previous observations in Wistar (Ignatowska-Jankowska et al. 2011) and Sprague Dawley rats (Henderson et al. 2023), to the ZDF model for the first time.

ZDF rats tend to develop insulin resistance and hyperglycemia as early as 10–15 weeks of life (Al-Awar et al. 2016). ZDF animals in our experiment were not provided antidiabetic or insulin treatment, thus, were maintained in uncontrolled diabetes and reached ~20 mmol/l fasting glucose, which CBD treatment failed to mitigate (Fig. 3).

Literature on the glycemic effects of CBD is ambiguous: some studies report CBD to decrease (Bielawiec et al. 2020), others to increase fasting insulin (Zorzenon et al. 2019). Effect of CBD on glucose levels are also contradictory as studied in various (Wistar and Sprague Dawley) rat models (Chaves et al. 2020; Santiago et al. 2019; Reyes-Cuapio et al. 2021; Hopkins et al. 2023). Our findings confirm that CBD does not improve glycemic control in the ZDF model. In light of this, the observed effects of CBD on ZDF rats appear to be independent of glucose-related metabolic pathways, a novel observation in this specific strain.

### Effects on electroretinogram

The effect of isolated CBD on the ZDF rat retina has not yet been studied using flash electroretinography. The following reviews provide a good summary of available data in this field (Wang and Danesh-Meyer 2021; Zantut et al. 2020). Prior human studies on cannabis consumers reported increased background noise on the electroretinogram (Lucas et al. 2019), but this study failed to isolate the specific role of CBD from other compounds naturally found in hemp (Cerino et al. 2021). According to our results, diabetic state and CBD treatment altered the electroretinogram statistically significantly.

Dark-adapted measurements in general reflect the functionality of mainly rods (a-wave), and rod-secondary cells (b-wave), especially bipolars, with the addition that brighter dark-adapted stimuli elicit mixed rod and cone (a-wave) and mixed rod-secondary and cone-secondary (b-wave) cell activity (Nixon et al. 2001). Light-adapted b-waves (in rats, light-adapted a-waves may not always be clearly discernible and suitable for reliable analysis, as they are potentially masked by larger b-waves according to the literature (Nixon et al. 2001; Bui and Fortune 2006)) reflect electrical activity of cone-secondary cells, since the background light saturate rods (Calcagni et al. 2024).

#### Dark-adapted ERG results

Our results indicate that the diabetic state slowed down photoreceptor electric function, i.e. prolonged dark-adapted a-wave implicit times (Fig. 5), consistent with observations by Johnson et al. in 22-week-old ZDF rats (Johnson et al. 2013). In the present study CBD was unable to improve this delay, further elongation was observed. A hypothesis might be impaired photoreceptor kinetics: either the pathway to closure of cGMP-mediated Na+-channels (“eliciting time”), or the pathway to generate electric activity (the closure itself) is more sluggish (“pathway time”). While these two specific kinetic components remain to be isolated, degeneration of retinal neurons associated with chronic diabetes remains the most plausible explanation for these findings (Tan and Wong 2022).

In contrast to the reduced a-wave amplitudes we measured in aged, 68-week-old ZDF rats (Wachal et al. 2021), the present study found higher dark-adapted a-wave amplitudes in 7-month-old ZDF animals compared to Lean controls, consistent with 22-week-old ZDF rats (Johnson et al. 2013). CBD-treated ZDF rats exhibited even greater amplitudes specifically at high stimulus strength in our current study. While one hypothesis could be retinal cell survival supported by the observed reduction in edema (please see 'Histological changes' section), immunohistological cell counting was not performed. Another hypothesis might be based on CBD-mediated inhibition of glutamate release and subsequent photoreceptor hyperpolarization (El-Remessy et al. 2003; Peres et al. 2018) leading to the highest a-wave amplitudes measured in the ZDF+CBD group. Although the mechanism remains to be elucidated, this study provides novel evidence that CBD treatment can improve the amplitude while slowing the kinetics of dark-adapted a-waves.

Dark-adapted b-waves generally showed greater amplitudes in CBD-treated ZDF animals compared to Lean. This is consistent with the increased amplitudes observed in case of a-waves. Since higher b-wave amplitudes occurred even at light stimulus strengths where a-wave amplitudes did not increase, CBD-elicited improved survival of post-receptoral cells may be considered as a hypothesis. Again a more plausible hypothesis is related to the decreased release of glutamate from photoreceptors resulting in depolarization of second-order neurons. While untreated ZDF animals exhibited b-wave changes consistent with diabetic retinal degeneration (Tan and Wong 2022), CBD-treated rats showed both significantly higher amplitudes and shorter implicit times compared to Lean controls. A hypothesis might be that CBD may accelerate and enhance post-receptoral cell activation, potentially through modulated glutamatergic signaling. A novel finding of this study is that dark-adapted b-waves in CBD-treated ZDF animals exhibited significantly higher amplitudes and shorter implicit times compared to the Lean control group.

#### Light-adapted ERG results

Results of light-adapted ERG further clarify the effect of CBD since cone-elicited secondary responses in ZDF+CBD group were reduced and delayed compared to Lean (Fig. 8). Amplitudes of light-adapted b-waves did not differed significantly from untreated ZDF, which means that CBD was not able to enhance cone-secondary electrical activity. Consequently, the elevated mixed rod-cone dark-adapted b-wave responses likely stem from CBD-mediated increases in the electrical activity of rod-driven secondary cells, marking a novel result of this study.

Light-adapted electroretinogram of ZDF animals showed smaller and sometimes slower b-waves, likely reflecting diabetic retinal degeneration (Tan and Wong 2022) and a reduced number of cone-secondary cells. This is further corroborated by our Histology results, which showed greater edema in ZDF group. Although, without immunohistochemistry we cannot rule out the hypothesis that perhaps the same amount of cone-secondary cells function less efficiently, which CBD was unable to counterbalance. In the literature light-adapted ERG measurements of ZDF animals are scarce and published results are quite inconsistent. For instance, fluctuating light-adapted b-wave amplitudes were noted by Johnson et al. depending on the developmental stage (Johnson et al. 2013). Nevertheless, in case of 22 weeks old ZDF rats, the age group most comparable to our 7-month-old animals, significantly prolonged implicit times and (non-significantly) diminished amplitudes were measured by them, aligning with our present findings. Light-adapted ERG results indicate that CBD did not significantly alter signal transmission kinetics along the cone photoreceptor to cone bipolar cell pathway, a novel observation in this model.

#### Oscillatory potentials and flicker ERG results

While decreased oscillatory potential (OP) amplitudes are hallmarks of human diabetes (Midena et al. 2021), rodent models show inconsistent trends: sometimes no change (Hancock and Kraft 2004; Ablonczy et al. 2018), or even increased OP amplitudes (Allen et al. 2023) may be observed. A characteristic feature of diabetes, even in rats, is an elongated OP implicit time, which was also shown in our study (Fig. 10). We observed increased dark-adapted and decreased light-adapted OP amplitudes in the diabetic group; however, CBD treatment failed to reverse these alterations. This study provides the first evidence that CBD does not mitigate diabetes-induced changes in OP amplitudes or implicit times in the ZDF model.

Interestingly however, CBD demonstrated a variability-lowering property of both dark-adapted and light-adapted OP amplitudes (Fig. 13). Violin plots reveal that while untreated ZDF rats exhibited a bimodal (two-poled) distribution of individual amplitudes, distribution of the most likely variants of OP amplitudes (the widest part of the violin) of ZDF+CBD and Lean groups are comparable. Consistent with this the OP amplitude variability is higher in ZDF group, while CBD treatment decreased it in the diabetic animals, which is a novel result. Considering the high intra- and intersubject variability typically associated with (human) OP amplitudes, some authors came to the conclusion, that instead of OP amplitudes, OP implicit times could be better indicators of retinopathy (Kothe et al. 1989; Polo et al. 1995). Similarly to OP implicit times, our findings suggest that OP amplitude variability may serve as a more sensitive diagnostic marker for early retinopathy than absolute amplitudes. If proven to be a sensitive indicator of diabetes or therapeutic response, OP variability could potentially detect retinal changes even before amplitude changes appear. However, further research is required.

Flicker ERG was employed to assess cone-driven responsiveness under rod-saturating conditions using stroboscopic stimuli, to determine the ability of the retina to react to rapid light flashes with the same frequency (McAnany et al. 2022; Kondo and Sieving 2001). Similarly to OPs, variability of flicker peak intervals was calculated. Untreated ZDF rats exhibited more scattered flicker peak mean intervals and and higher flicker interval variability compared to Lean controls. CBD was demonstrated to decrease flicker peak interval variability, which is a novel result. Similarly to OP variability, flicker interval variability may represent a potential novel diagnostic tool in retinal electrophysiology. While these results are promising, further studies are required to validate their clinical utility.

### Histological changes

Histology results are shown in Fig. 15. According to our measurements CBD was able to significantly decrease diabetes-related thickening of each retinal layer of ZDF animals, which is a novel result. This finding aligns with our previous work with sour cherry seed extract, which similarly reduced retinal edema in ZDF rats (Varga et al. 2017), and supports the potential of herbal compounds in mitigating retinal ischemia-reperfusion injury shown in other rat models (He et al. 2014; Huang et al. 2015; Han et al. 2020; Tan et al. 2017).

Although untreated ZDF rats exhibited increased retinal thickness and higher ERG amplitudes, these findings do not appear to stem from an increased cell number. On the contrary, edema is a documented consequence of ischemia (Kaur et al. 2008) and a prognostic marker of retinal cell death (Kaur et al. 2008; Song et al. 2025). And ischemia is a proven pathomechanism of diabetes (Tan and Wong 2022; Gettinger et al. 2025). Conversely, CBD-mediated reduction in tissue edema likely promotes retinal cell survival, potentially underlying the enhanced dark-adapted ERG amplitudes. While due to the lack of immunohistochemical cell counting, a limitation of the study, this correlation remains a hypothesis, our findings provide a strong basis for further exploring CBD’s therapeutic potential in diabetic retinopathy. 

## Conclusion

Our study demonstrates that CBD exerts functional retinoprotective effects in ZDF rats through a glucose-independent mechanism, normalizing weight gain and preventing retinal thickening. CBD selectively enhanced rod-mediated (dark-adapted) ERG amplitudes without affecting the cone pathway, and reduced variability in OPs and flicker intervals. We introduce OP amplitude variability and flicker interval variability as two novel indicators of early retinal dysfunction and therapeutic response. Our results highlight the therapeutic potential of CBD in diabetic retinopathy and warrant further research.

## Supplementary Information

Supplementary Material 1.

## Data Availability

Dataset available on request from the authors, however restrictions may apply as the datasets presented in this article are the property of the University of Debrecen. Requests to access the datasets should be directed to the authors.

## Abbreviations

CBD: Cannabidiol
ERG: Electroretinography
GCL: Ganglion Cell Layer
ILM: Inner Limiting Membrane
INL: Inner Nuclear Layer
IPL: Inner Plexiform Layer
OGTT: Oral Glucose Tolerance Test
OLM: Outer Limiting Membrane
ONL: Outer Nuclear Layer
OP: Oscillatory Potential
OPL: Outer Plexiform Layer
PL: Photoreceptor Layer
SD: Standard Deviation
SEM: Standard Error of the Mean
THC: Tetrahydrocannabinol
ZDF: Zucker Diabetic Fatty
